# ^68^Ga-TRAP-(RGD)_3_ Hybrid Imaging for the *In Vivo* Monitoring of α_v_ß_3_-Integrin Expression as Biomarker of Anti-Angiogenic Therapy Effects in Experimental Breast Cancer

**DOI:** 10.1371/journal.pone.0168248

**Published:** 2016-12-19

**Authors:** Philipp M. Kazmierczak, Andrei Todica, Franz-Josef Gildehaus, Heidrun Hirner-Eppeneder, Matthias Brendel, Ralf S. Eschbach, Magdalena Hellmann, Konstantin Nikolaou, Maximilian F. Reiser, Hans-Jürgen Wester, Saskia Kropf, Axel Rominger, Clemens C. Cyran

**Affiliations:** 1 Institute for Clinical Radiology, Laboratory for Experimental Radiology, Ludwig-Maximilians-University Hospital Munich, München, Germany; 2 Department of Nuclear Medicine, Ludwig-Maximilians-University Hospital Munich, München, Germany; 3 Department of Diagnostic and Interventional Radiology, University Hospital Tübingen, Tübingen, Germany; 4 Lehrstuhl für Pharmazeutische Radiochemie, Technical University Munich, München, Germany; 5 SCINTOMICS GmbH, Fürstenfeldbruck, Germany; Universidade de Sao Paulo, BRAZIL

## Abstract

**Objectives:**

To investigate ^68^Ga-TRAP-(RGD)_3_ hybrid imaging for the in vivo monitoring of α_v_ß_3_-integrin expression as biomarker of anti-angiogenic therapy effects in experimental breast cancer.

**Materials and Methods:**

Human breast cancer (MDA-MB-231) xenografts were implanted orthotopically into the mammary fat pads of n = 25 SCID mice. Transmission/emission scans (53 min to 90 min after i.v. injection of 20 MBq ^68^Ga-TRAP-(RGD)_3_) were performed on a dedicated small animal PET before (day 0, baseline) and after (day 7, follow-up) a 1-week therapy with the VEGF antibody bevacizumab or placebo (imaging cohort n = 13; therapy n = 7, control n = 6). The target-to-background ratio (TBR, VOImax_tumor_/VOImean_muscle_) served as semiquantitative measure of tumor radiotracer uptake. Unenhanced CT data sets were subsequently acquired for anatomic coregistration and morphology-based tumor response assessments (CT volumetry). The imaging results were validated by multiparametric ex vivo immunohistochemistry (α_v_ß_3_-integrin, microvascular density–CD31, proliferation–Ki-67, apoptosis–TUNEL) conducted in a dedicated immunohistochemistry cohort (n = 12).

**Results:**

^68^Ga-TRAP-(RGD)_3_ binding was significantly reduced under VEGF inhibition and decreased in all bevacizumab-treated animals (ΔTBR_follow-up/baseline_: therapy -1.07±0.83, control +0.32±1.01, p = 0.022). No intergroup difference in tumor volume development between day 0 and day 7 was observed (Δvolume_therapy_ 134±77 μL, Δvolume_control_ 132±56 μL, p = 1.000). Immunohistochemistry revealed a significant reduction of α_v_ß_3_-integrin expression (308±135 vs. 635±325, p = 0.03), microvascular density (CD31, 168±108 vs. 432±70, p = 0.002), proliferation (Ki-67, 5,195±1,002 vs. 7,574±418, p = 0.004) and significantly higher apoptosis (TUNEL, 14,432±1,974 vs. 3,776±1,378, p = 0.002) in the therapy compared to the control group.

**Conclusions:**

^68^Ga-TRAP-(RGD)_3_ hybrid imaging allows for the in vivo assessment of α_v_ß_3_-integrin expression as biomarker of anti-angiogenic therapy effects in experimental breast cancer.

## Introduction

Hybrid imaging has evolved into an essential diagnostic modality for state-of-the-art tumor staging in modern clinical oncology, allowing for a non-invasive tumor characterization on the morphological, functional, and molecular level [1]. While ^18^F-fluorodeoxyglucose (FDG) remains the most widely applied tracer in positron emission tomography (PET), the arsenal of diagnostic radiotracers has been expanded significantly over the last decade [2–5]. The development of radiolabeled arginylglycylaspartic acid (RGD) tracers has enabled the specific targeting of α_v_ß_3_-integrin, an endothelial and tumor cell receptor with a significant role in neoangiogenesis [6]. Integrins are a family of transmembrane proteins interacting with a variety of ligands from the extracellular matrix [7]. Mediating cell adhesion and extracellular-to-intracellular signaling pathways, they have been identified as key players in tumor progression and metastasis [7]. α_v_ß_3_-integrin is overexpressed by angiogenic endothelium and tumor cells and is involved in various angiogenic signaling cascades including the Vascular Endothelial Growth Factor (VEGF) pathway [8, 9]. VEGF augments endothelial cell migration and adhesion by indirect, receptor-mediated α_v_ß_3_-integrin activation [9]. Vice versa, inhibition of VEGF was shown to significantly suppress tumor α_v_ß_3_-integrin expression in line with a significant reduction of microvascular density [10–12]. α_v_ß_3_-integrin is therefore proposed a marker of angiogenic activity and a target structure for the in vivo imaging of tumor neoangiogenesis [8]. Inhibition of the VEGF pathway using the VEGF antibody bevacizumab as either single or combination therapy has become an established clinical anti-angiogenic treatment regimen applied in various tumor entities, including non-small-cell lung, colorectal, and breast cancer [13]. In vivo, intact murine VEGF is not recognized by bevacizumab, while cleaved murine VEGF can be recognized by this antibody in Western Blots [14].

However, a recent study confirmed the anti-VEGF activity of bevacizumab in murine models [15]. Accordingly, Wang and colleagues recently demonstrated that bevacizumab exhibits anti-VEGF activity in human colorectal carcinoma xenografts in mice [16].

RGD radiotracers have been shown to allow for the non-invasive quantification of α_v_ß_3_-integrin expression in vivo [6]. Semiquantitative measures of tissue RGD radiotracer uptake demonstrated excellent correlations with the α_v_ß_3_-integrin receptor expression as quantified by ex vivo immunohistochemistry [17, 18]. The selective in vivo imaging of endothelial α_v_ß_3_-integrin expression as surrogate of tumor angiogenesis may be limited by α_v_ß_3_-integrin overexpressed on tumor cell surfaces [10, 12]. The triple-negative human breast cancer cell line MDA-MB-231 exhibits only marginal tumor cell α_v_ß_3_-integrin expression and therefore potentially facilitates the in vivo imaging of α_v_ß_3_-integrin expressing tumor vasculature [10, 19]. However, tumors in which α_v_ß_3_-integrin expression remains predominantly reserved to the endothelium require RGD radiotracers with a high target affinity, as whole-tumor α_v_ß_3_-integrin expression and therefore whole-tumor radiotracer uptake will remain relatively low. The novel cyclic radiotracer ^68^Ga-1,4,7-triazacyclononane-1,4,7-tris[(2-carboxyethyl)(methylenephosphinic acid]) (TRAP)-(RGD)_3_ may prove advantageous for the in vivo imaging of tumor models with a low overall α_v_ß_3_-integrin expression such as MDA-MB-231, as it demonstrated a more than seven-fold higher in vitro target affinity compared to the monomeric radiotracers ^18^F-Galacto-RGD and ^68^Ga-1,4,7-triazacyclononane-1,4-bis(acetic acid)(NODAGA)-RGD [20].

The aim of the present study was to investigate the applicability of ^68^Ga-TRAP-(RGD)_3_-PET/CT for the quantitative and longitudinal in vivo imaging of a low α_v_ß_3_-integrin expressing human breast cancer model. We therefore hypothesized that ^68^Ga-TRAP-(RGD)_3_-PET/CT allows for the in vivo monitoring of α_v_ß_3_-integrin expression as biomarker of anti-angiogenic therapy effects in orthotopic MDA-MB-231 breast cancer xenografts in mice treated with the VEGF antibody bevacizumab over the course of one week.

## Materials and Methods

The study was approved by the Government of Upper Bavaria Committee of Animal Research (Gz.: 55.2-1-54-2532-82-2014). It was conducted in accordance with the National Institute of Health Guide for the Care and Use of Laboratory Animals. All efforts were taken to minimize animal suffering. The mice were kept in individually-ventilated cages (n = 4 per cage; constant temperature 26°C, relative air humidity 65%, 18 room air changes per hour, light-dark-cycle 12 hours). Animals were nourished ad libitum with water and dedicated small animal nutrition. In order to assure environmental enrichment, nest boxes and nestlets were provided. After tumor cell inoculation, animals were monitored once a day and tumor growth was determined using a caliper. Weight and tumor growth were noted on a daily basis. Abnormal inactivity was considered to indicate pain and was treated by analgesia (buprenorphin 0.5 mg/kg body weight s.c.). Imaging was performed under isoflurane anesthesia (2.5% in 1.0 L 100% O_2_/min for induction and 1.5% in 1.0 L 100% O_2_/min for maintenance, respectively). The animals were euthanized if one of the following conditions were present: no tumor growth, tumor size >1.5 cm, ulceration of tumors, weight loss >20%, apathy, remarkable defense reaction when tumors were palpated, remarkable breathing difficulties, lameness, and a non-physiologic body posture. Animals were euthanized under inhalation anesthesia (5.0% isoflurane in pure oxygen) by intracardial injection of a saturated potassium chloride solution.

### Tumor model and study setup

In a previous study, we confirmed the low tumor cell α_v_ß_3_-integrin expression of the investigated MDA-MB-231 (ATCC^®^ HTB-26^™^, Manassas, VA) cell line by flow cytometry experiments [10]. 3 x 10^6^ MDA-MB-231 cells were resuspended in 0.05 mL of 1:1 Matrigel^™^ (BD Biosciences, San Jose, CA) / phosphate-buffered saline (PBS) solution and injected into the mammary fat pads of n = 25 Severe Combined Immunodeficiency (SCID) mice (age: 7 to 8 weeks; Harlan Laboratories Inc., Indianapolis, IN). After reaching a tumor size of 0.5 cm, the animals were assigned to either the imaging (n = 13) or the immunohistochemistry cohort (n = 12).

Imaging cohort (n = 13): The animals were randomly assigned to either the therapy (n = 7) or the control group (n = 6). After the baseline scan (day 0), animals were treated daily with either bevacizumab (therapy group; Avastin^®^, Hoffmann-La Roche AG, Basel, Switzerland; 5 mg/kg body weight) or a volume-equivalent placebo solution (control group; 0.9% sodium chloride) for one week (days 1 to 6). The therapeutic agents were injected into the peritoneal cavity using a 27-gauge needle. On day 7, the follow-up imaging was performed.

**Fig 1:**
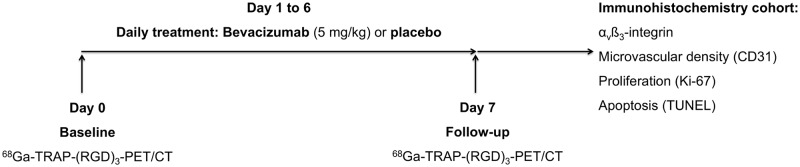
Study setup. After the ^68^Ga-TRAP-(RGD)_3_-PET/CT baseline scan (day 0), animals of the imaging cohort were treated daily with either bevacizumab (therapy group) or a volume-equivalent placebo solution (control group) for 6 days. ^68^Ga-TRAP-(RGD)_3_-PET/CT follow-up scan was performed on day 7. Animals of the immunohistochemistry cohort were randomized to a therapy and a control group and treated analogously to the imaging cohort with either bevacizumab (therapy group) or placebo (control group) for 6 days. On day 7, the animals of the immunohistochemistry cohort were sacrificed and the tumors were explanted in order to undergo immunohistochemical workup with regard to α_v_ß_3_-integrin expression, microvascular density (CD31), proliferation (Ki-67), and apoptosis (TUNEL).

Immunohistochemistry cohort (n = 12): In order to exclude competitive blocking effects between the primary anti-α_v_ß_3_-integrin antibody used for the immunohistochemical staining and receptor-bound ^68^Ga-TRAP-(RGD)_3_, the ex vivo immunohistochemical validation was conducted in a separate animal cohort. Analogously to the imaging cohort, mice were randomly assigned to either the therapy (n = 6) or the control group (n = 6), and the above-described one-week therapy regimen was carried out accordingly. On day 7, the animals were sacrificed and tumors were explanted and cut in half in order to undergo the immunohistochemical workup. One half was cryopreserved in liquid nitrogen at -196°C for 1 min and then stored at -80°C, while the other half was fixed in formalin. Fig 1 provides an overview of the study setup.

### ^68^Ga-TRAP-(RGD)_3_

^68^Ga-TRAP-(RGD)_3_ was synthesized as described previously [20–22] with slight modifications. Briefly, 25 μg of the precursor (AVEBETRIN, SCINTOMICS GmbH, Fürstenfeldbruck, Germany) dissolved in 288 μL 1 M Na-acetate were labelled with 300–400 MBq ^68^GaCl_3_ eluted in 2 mL 0.1 N HCl from a ^68^Ge/^68^Ga-generator (IGG100-50M, Eckert & Ziegler Radiopharma GmbH, Berlin, Germany). After heating for 10 min at 90°C followed by purification via C18 cartridge the final injection solution was prepared by adjusting the pH with phosphate buffer to 7.2. Radiochemical purity of each preparation was >98% determined by radio-high performance liquid chromatography. The in vivo properties of ^68^Ga-TRAP-(RGD)_3_ in mice were characterized previously, including blocking, metabolite, and biodistribution studies [20].

### Imaging protocol

Imaging was performed under isoflurane anesthesia (2.5% in 1.0 L 100% O_2_/min for induction and 1.5% in 1.0 L 100% O_2_/min for maintenance, respectively). ^68^Ga-TRAP-(RGD)_3_ (20 MBq in a total volume of 100 μL) was injected via the lateral tail vein. The animals were then placed within the aperture of a dedicated small animal PET (Inveon Dedicated PET, Preclinical Solutions, Siemens Healthcare Molecular Imaging, Knoxville, TN) and a 7-minute transmission scan employing a rotating ^57^Co source was started 53 min after injection. Transmission scans were acquired for scatter and attenuation correction. Subsequently, an emission scan (three-dimensional listmode acquisition) was initiated from 60 to 90 min after radiotracer injection. An unenhanced CT (Somatom Force, Siemens Healthcare, Erlangen, Germany; 35 mAs, 100 kV, slice thickness 0.6 mm; reconstructed in axial, coronal, and sagittal planes) data set in identical animal position relative to the PET scan was acquired, serving for anatomic coregistration, tumor localization, and morphology-based assessments of tumor response (CT volumetry at day 0 and day 7).

### Data post-processing and analysis

**Fig 2:**
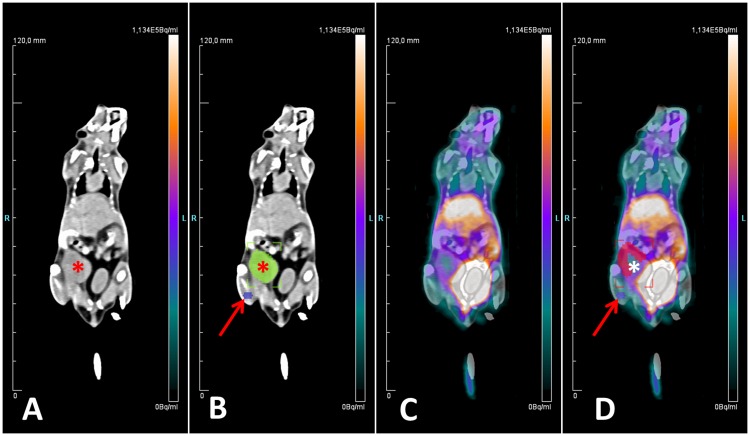
VOI selection. Images in coronal reconstruction. A: unenhanced CT (tumor indicated by asterisk); B: unenhanced CT with tumor (green, asterisk) and muscle (violet, arrow) VOIs; C: fused PET and CT data sets; D: fused PET and CT data sets with tumor (pink, asterisk) and muscle (violet, arrow) VOIs. Tumor and muscle VOIs selected on the unenhanced CT (B) were superimposed on the PET data sets (D) to allow for a quantification of the PET signal. Note that tumor regions with significant signal spillover from the urinary bladder were excluded from the quantitative analysis (D).

The PET data sets were analyzed using dedicated post-processing software (Inveon Acquisition Workplace, Siemens Medical Solutions, Knoxville, TN). Data sets were reconstructed as static images using OSEM 3D and MAP 3D algorithms with 4 and 32 iterations, respectively. PET image parameters were as follows: 256 x 256 matrix, 159 slices, slice thickness 0.796 mm, zoom factor 100%, spatial resolution 1.5 mm. PET data sets were normalized and corrected for attenuation and scatter as well as for randoms, dead time, and decay. CT Volumes-of-Interest (VOI) were superimposed on PET images to determine corresponding volumes for quantification of the PET signal. Fig 2 provides details on VOI selection. The target-to-background ratio (TBR, Volume-of-Interest (VOImax_tumor_/VOImean_muscle_) was determined as semiquantitative measure of tumor radiotracer accumulation before (day 0) and after treatment (day 7). As the radiotracer uptake is normalized to a reference tissue (muscle), TBR is, compared to the standardized uptake value, less sensitive to inter- and intraindividual variations in mouse weight, radiotracer blood clearance, and radiotracer dose. Tumor regions with spillover from the urinary bladder were excluded from the analysis. Tumor volumes [μL] at baseline and follow-up were measured by CT volumetry using the above-mentioned post-processing software. PET and CT data sets were analyzed coregistered as side-by-side examinations.

### Immunohistochemistry

#### α_v_β_3_-integrin

After fixation in acetone at a temperature of -20°C and blocking of endogenous peroxidase (peroxidase blocking reagent; DAKO, Hamburg, Germany), tumor cryosections (6 μm) were incubated with the primary mouse anti-human integrin α_v_β_3_ monoclonal antibody (anti-human Integrin αvβ3 LM609, 1:50; Merck Millipore, Darmstadt, Germany) for 2 h. The tissue sections were subsequently treated with a secondary anti-mouse antibody (Envsion^™^+ Kit; DAKO, Hamburg, Germany). α_v_β_3_-integrin expression was then visualized using an AEC-Chromogen (Envsion^™^+ Kit; DAKO, Hamburg, Germany).

#### CD31, Ki-67, TUNEL

To assess microvascular density (CD31), tumor cell proliferation (Ki-67), and apoptosis (TUNEL), formaldehyde-fixed and paraffin-embedded tissue sections were dewaxed and rehydrated using standard protocols. Expression of CD31 and Ki-67 were analyzed using the Envsion^™^+System-HRP AEC/DAB system (DAKO, Hamburg, Germany) according to the manufacturer’s instructions. After microwave antigen-demasking in a target-retrieval solution (DAKO, Hamburg, Germany) and peroxidase blocking, tissue specimens were incubated with the primary anti-CD31 (1:50; ab28364, Abcam, Cambridge, UK) or anti-Ki-67 (1:100; ab16667, Abcam, Cambridge, United Kingdom) antibodies overnight. Subsequently, tissue sections were treated with a secondary anti-rabbit antibody (Envsion^™^+ Kit; DAKO, Hamburg, Germany) and expression of CD31/Ki-67 was visualized using an AEC-chromogen (CD31) or a DAB-chromogen (Ki-67), respectively. Apoptosis was assessed by TUNEL assays (In situ Cell Death Detection Kit; Sigma-Aldrich, Taufkirchen, Germany). Fluorescence microscopy at a standard fluorescent filter (520±20 nm; DM 2500, Leica Camera AG, Wetzlar, Germany) was used to quantify the number of apoptotic cells.

Quantitative immunohistochemical parameters were expressed as the number of positively stained microvessels (α_v_β_3_-integrin and CD31) or cells (Ki-67 and TUNEL) in ten random high-power fields at 200x magnification.

#### CD31/α_v_β_3_-integrin fluorescent double staining

Cryosections were prepared, fixed in acetone at a temperature of -20°C for 10 min, air-dried for 20 min, and unspecific binding sites were blocked with normal goat serum (5% in 1x PBS) for 1 h. Tissue sections were then treated with the primary anti-CD31 (1:50; Abcam, Cambridge, United Kingdom) and anti-α_v_β_3_-integrin (LM609: 1:50, Merck Millipore, Darmstadt, Germany) antibodies at 4°C overnight. After incubation with dye-conjugated secondary antibodies (goat anti-Mouse IgG Alexa Fluor 488: 1:500; Abcam, Cambridge, United Kingdom; donkey anti-Rabbit Cy3^™^: 1:300; Jackson ImmunoResearch Inc., West Grove, PA) and nuclear staining (DAPI; 1 μg/mL; Roth, Karlsruhe, Germany), tissue sections were mounted and coverslipped with Fluoromount^™^ (Sigma, Taufkirchen, Germany). Images were analyzed using a dedicated fluorescence microscope (DM 2500; Leica Camera AG, Wetzlar, Germany).

### Validation of ^68^Ga-TRAP-(RGD)_3_ binding specificity in MDA-MB-231 xenografts using autoradiography and immunofluorescence imaging

Target specificity of ^68^Ga-TRAP-(RGD)_3_ was validated in two steps. First, we conducted autoradiography experiments of radiotracer-incubated tumor sections with and without blocking of the α_v_β_3_-integrin receptor using a specific antibody (step 1). Second, the specific α_v_β_3_-integrin receptor blocking in the autoradiography experiments was confirmed by secondary immunofluorescence stainings (step 2).

Step 1: For autoradiography, tumor cryosections (6 μm) mounted on glass slides were unfrozen and dried at room temperature for 2 h. Half number of the slide-mounted tumor sections were washed with binding buffer (Tris-HCl 50 mM, pH 7.4) and air-dried for 1.5 h. For the blocking experiments, the remaining half of the slide-mounted tumor sections were incubated with the primary anti-α_v_β_3_-integrin antibody (LM609: 1:20, Merck Millipore, Darmstadt, Germany) for 1 h, then washed with binding buffer (Tris-HCl 50 mM, pH 7.4) and air-dried for 1.5 h. Subsequently, all slides (without and with α_v_β_3_-integrin receptor blocking) were incubated with ^68^Ga-TRAP-(RGD)_3_ (5 MBq in 50 mL Tris-HCl 50 mM), washed with binding buffer, and air-dried for 1 h. The slides were then placed on autoradiography imaging plates (BAS cassette2 2025 imaging plates, Fujifilm, Tokyo, Japan) for 2.5 h, and imaging plates were subsequently scanned by autoradiography (CR-35-BIO, 25 μm resolution, Elysia-Raytest GmbH, Straubenhardt, Germany). Image analysis was performed using dedicated post-processing software (AIDA image analyzing software V4.50, Elysia-Raytest GmbH, Straubenhardt, Germany). Signal intensity (intensity per area; background subtraction) was extracted in adjacent blocked and unblocked tumor sections. The ratio between unblocked and blocked tumor sections was calculated.

Step 2: In order to validate the specific α_v_β_3_-integrin receptor blocking in the autoradiography experiments, we performed α_v_β_3_-integrin fluorescent stainings as described above. Briefly, the same tumor sections used for the autoradiography were incubated with the primary anti-α_v_β_3_-integrin (LM609: 1:50, Merck Millipore, Darmstadt, Germany) and dye-conjugated secondary antibodies (goat anti-Mouse IgG Alexa Fluor 488: 1:500; Abcam, Cambridge, United Kingdom; donkey anti-Rabbit Cy3^™^: 1:300; Jackson ImmunoResearch Inc., West Grove, PA) immediately after step 1. The tumor slides were then analyzed using a preclinical optical imaging system (In-Vivo FX PRO, Bruker Corp., Billerica, MA) with a charge-coupled device camera (excitation filter 480 nm, emission filter 535 nm, field-of-view 200 mm, focal plane 11 mm, 2x x-binning, 2x y-binning, 130 x 130 ppi). Fluorescent stainings were analyzed visually and interpreted qualitatively.

### Statistical analysis

The statistical analysis was performed using SPSS 23 for Windows (IBM Corp., Armonk, NY). Descriptive statistical data were expressed as arithmetic means with standard deviations at 95% confidence intervals. Wilcoxon/Mann-Whitney U tests were applied for intra- and inter-group comparisons of the quantitative parameters. Linear correlations between non-normally distributed variables were assessed by Spearman’s test. Autoradiographic signal intensities in unblocked and adjacent blocked tumor sections were compared using a paired student’s t test. Statistical significance was assumed for p-values <0.05.

## Results

### ^68^Ga-TRAP-(RGD)_3_ binding

**Fig 3:**
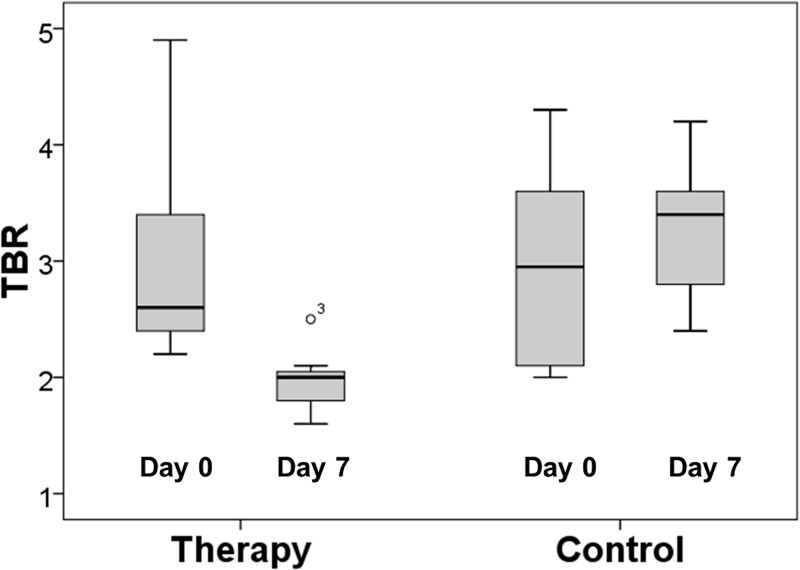
TBR at baseline and follow-up. Note the significant (p = 0.018) decrease in TBR in the therapy group between day 0 and day 7. No significant (p = 0.500) change in TBR was observed in the control group. There was no significant (p = 0.836) difference in baseline TBR between the therapy and the control group.

**Fig 4:**
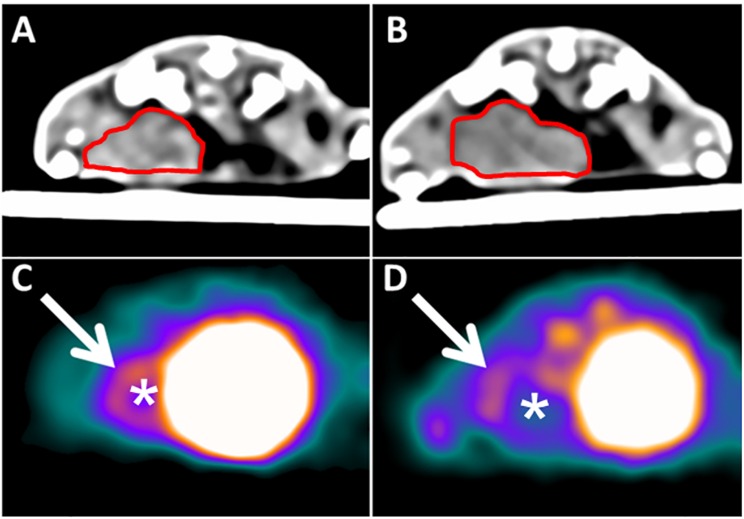
Representative coregistered ^68^Ga-TRAP-(RGD)_3_-PET/CT data sets of one animal from the therapy group. Animal in prone position. Top row: unenhanced CT data sets at baseline (A) and follow-up (B). The tumor placed in the left mammary fat pad is circled in red. Bottom row: PET data sets at baseline (C) and follow-up (D). White arrows and asterisks indicate the tumor. Note the significant decrease in tumor radiotracer uptake between baseline (day 0, C) and follow-up (day 7, D). The unenhanced CT data sets (A—baseline; B—follow-up) served for anatomic colocalization and allowed for the exclusion of relevant tumor necrosis.

^68^Ga-TRAP-(RGD)_3_ binding was significantly reduced following VEGF inhibition (mean ΔTBR_follow-up/baseline_: therapy -1.07±0.83, control +0.32±1.01; p = 0.022). We observed a significant decline of TBR in all animals of the therapy group (from TBR_therapy-baseline_ 3.04±0.95 to TBR_therapy-follow-up_ 1.97±0.29; p = 0.018). In the control group, TBR developed omnidirectionally, with an increase in n = 5 animals (from TBR_control-baseline_ 2.98±0.92 to TBR_control-follow-up_ 3.30±0.63; p = 0.500). There was no significant difference in baseline TBR between the therapy and the control group (TBR_therapy-baseline_ 3.04±0.95, TBR_control-baseline_ 2.98±0.92; p = 0.836). Table 1 provides individual TBR values of the animals of the therapy and the control group. TBR values of the therapy and control group are displayed as boxplots in Fig 3. Representative PET/CT images of a tumor before and after VEGF inhibition are shown in Fig 4.

**Table 1:** Imaging cohort: individual TBR values and tumor volumes for therapy and control group.

No.	Group (T/C)^a^	TBR_Baseline_	TBR_Follow-up_	ΔTBR	Vol_Baseline_ [μL]	Vol_Follow-up_ [μL]
1	T	2.5	2.0	-0.5	207	291
2	T	3.4	1.6	-1.8	527	739
3	T	4.9	2.5	-2.4	425	606
4	T	2.2	1.9	-0.3	193	430
5	T	2.3	2.1	-0.2	241	269
6	T	2.6	1.7	-0.9	51	157
7	T	3.4	2.0	-1.4	378	469
**mean±SD**^b^		3.04±0.95	1.97±0.29	-1.07±0.83^c^	289±162	423±203^d^
11	C	2.5	3.6	+1.1	182	298
12	C	2.0	2.4	+0.4	148	200
13	C	2.1	3.4	+1.3	194	364
14	C	4.3	2.8	-1.5	379	562
15	C	3.4	3.4	0	313	498
16	C	3.6	4.2	+0.6	273	356
**mean±SD**^b^		2.98±0.92	3.30±0.63	+0.32±1.01^c^	248±89	380±132^d^

^a^T = therapy, C = control;

^b^SD = standard deviation;

^c^p = 0.02 (therapy vs. control;

^d^p = 1.000 (Δvolume_therapy_ vs. Δvolume_control_)

### Tumor volume

Contrary to the changes in α_v_ß_3_-integrin expression detected by ^68^Ga-TRAP-(RGD)_3_-PET, there was no significant intergroup difference in tumor volume development over the course of the experiment (Δvolume_therapy_ 134±77 μL, Δvolume_control_ 132±56 μL; p = 1.000). There was no significant difference in baseline tumor volumes between therapy and control group (volume_therapy-baseline_ 289±162 μL, volume_control-baseline_ 248±89 μL; p = 0.628). No significant correlation between tumor volumes and TBR values at baseline and follow-up was observed (Spearman’s ρ -0.23; p = 0.261). Table 1 displays individual tumor volumes before and after treatment.

### Immunohistochemistry

**Fig 5:**
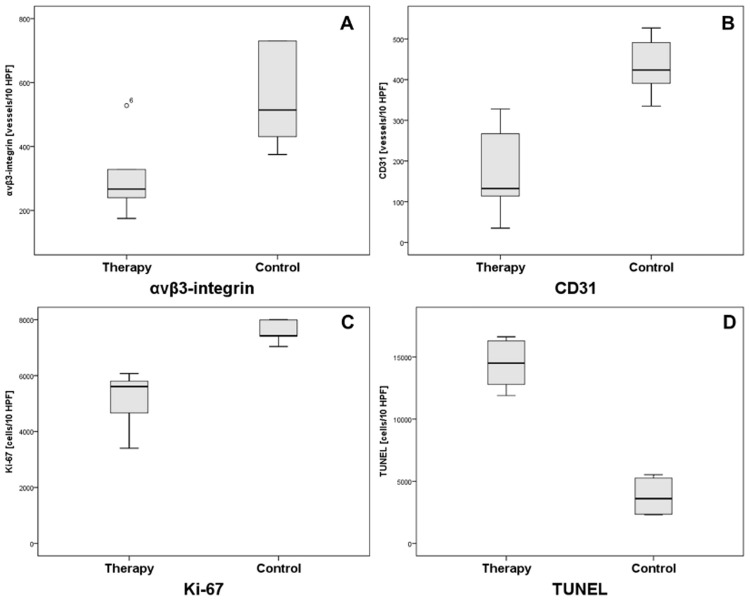
Quantitative immunohistochemical parameters for the therapy and control group. Note the significant (p = 0.03) suppression of α_v_ß_3_-integrin expression in the bevacizumab-treated group (A). Also note the significantly (p<0.01) lower microvascular density (CD31, B), proliferation (Ki-67, C), as well as the significantly (p = 0.002) higher apoptosis (TUNEL, D) in the therapy compared to the control group.

**Fig 6:**
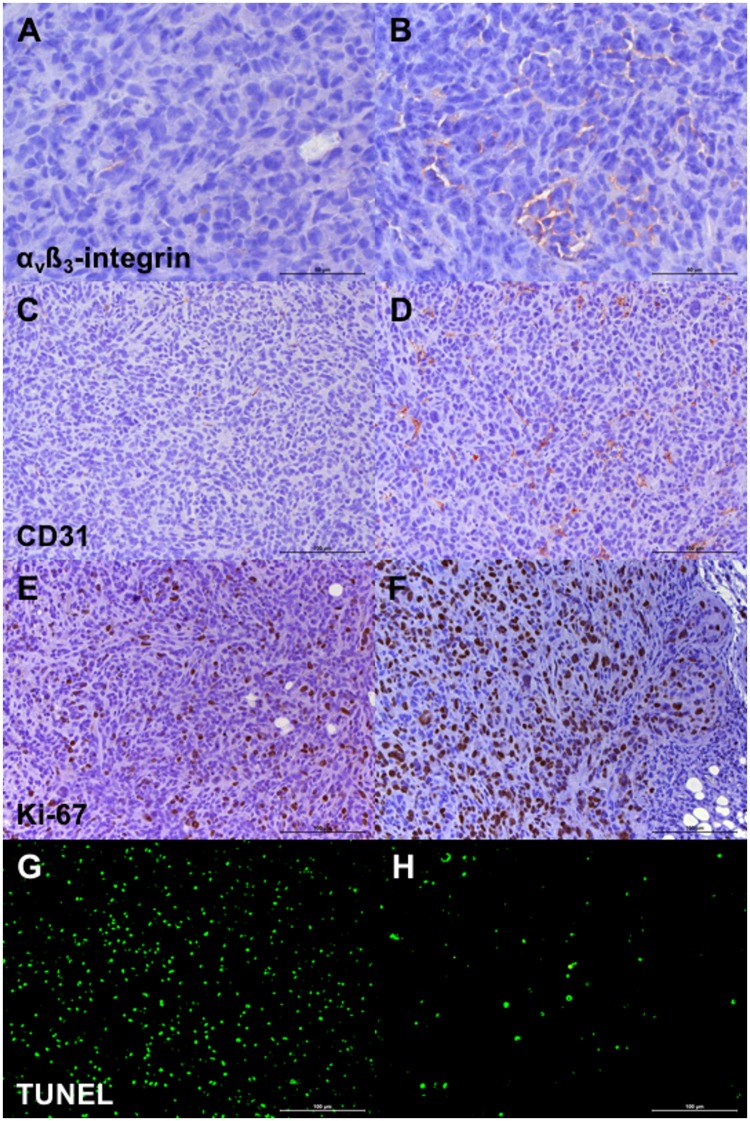
Representative tumor sections of the therapy and the control group. Note the lower α_v_ß_3_-integrin expression (A vs. B), microvascular density (CD31, C vs. D), proliferation (Ki-67, E vs. F) and the higher apoptosis (TUNEL, G vs. H) in the therapy compared to the control group

Immunohistochemistry revealed a significant reduction of α_v_ß_3_-integrin expression (308±135 vs. 635±325, p = 0.03), microvascular density (CD31, 168±108 vs. 432±70, p = 0.002), proliferation (Ki-67, 5,195±1,002 vs. 7,574±418, p = 0.004), as well as significantly higher apoptosis (TUNEL, 14,432±1,974 vs. 3,776±1,378, p = 0.002) in the therapy compared to the control group. Individual quantitative immunohistochemical parameters are summarized in Table 2. The immunohistochemical data are summarized by boxplots in Fig 5. Representative immunohistochemical tumor sections of the therapy and control group are shown in Fig 6.

**Table 2:** Immunohistochemistry cohort: individual values of immunohistochemical parameters for therapy and control group. Subsequent to follow-up imaging on day 7, tumors were explanted to undergo multiparametric immunohistochemistry with regard to α_v_ß_3_-integrin expression, microvascular density (CD31), proliferation (Ki-67), and apoptosis (TUNEL). Results are expressed as mean number of positively-stained microvessels (α_v_ß_3_-integrin and CD31) or cells (Ki-67 and TUNEL) per ten high-power fields at 200x magnification.

No.	Group (T/C)^a^	α_v_ß_3_-integrin	CD31	Ki-67	TUNEL
1	T	240	35	5,763	15,522
2	T	175	136	6,075	16,623
3	T	n/a	129	4,669	16,286
4	T	328	328	5,461	11,889
5	T	267	267	5,800	12,795
6	T	528	114	3,404	13,477
**mean ± SD**^b^		308±135	168±108	5,195±1,002	14,432±1,974
7	C	730	527	7,422	2,316
8	C	375	491	7,409	2,354
9	C	431	440	n/a	5,255
10	C	564	391	8,006	3,730
11	C	1,248	407	7,992	5,527
12	C	464	335	7,039	3,476
**mean ± SD**^b^		635±325^c^	432±70^d^	7,574±418^e^	3,776±1,378^f^

^a^T = therapy, C = control;

^b^SD = standard deviation;

^c^p = 0.03,

^d^p = 0.002,

^e^p = 0.004,

^f^p = 0.002 (therapy vs. control)

**Fig 7:**
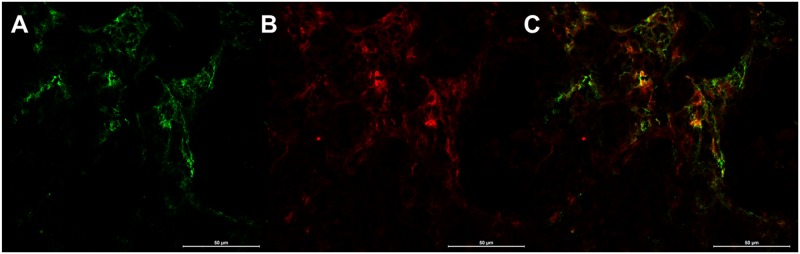
α_v_ß_3_-integrin/CD31 fluorescent double stainings. A— α_v_ß_3_-integrin; B—CD31; C—overlay of A and B. Fluorescent double stainings demonstrated a significant coexpression (C) of α_v_ß_3_-integrin and the endothelial receptor CD31 and therefore confirmed the predominantly endothelial expression of α_v_ß_3_-integrin in the investigated tumor model. No relevant tumor cell α_v_ß_3_-integrin expression was detected.

**Fig 8:**
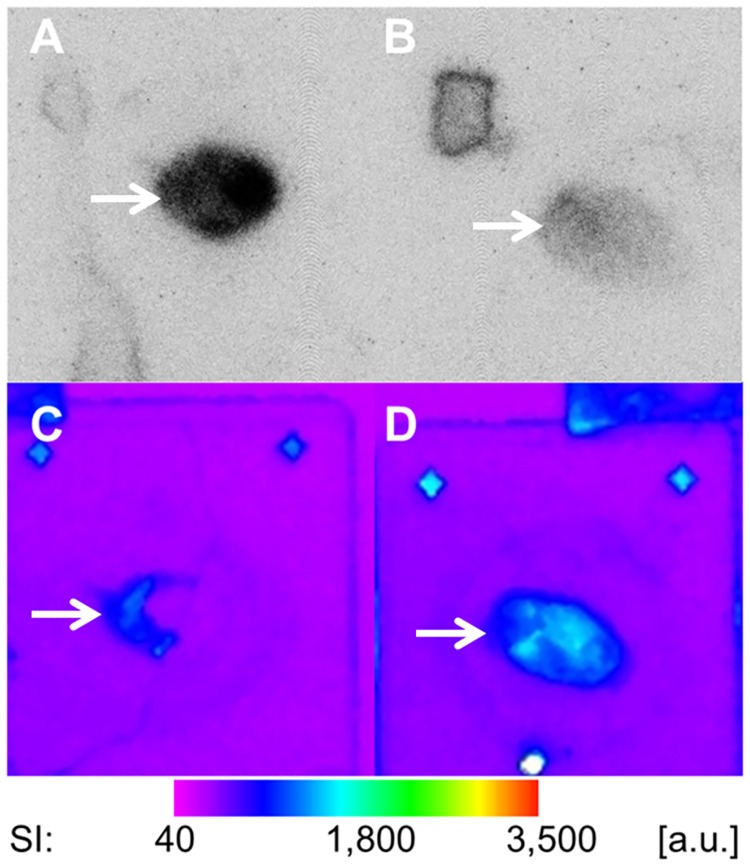
Validation of ^68^Ga-TRAP-(RGD)_3_ binding specificity in MDA-MB-231 human breast cancer xenografts. Top row (A and B): autoradiography of ^68^Ga-TRAP-(RGD)_3_-incubated MDA-MB-231 tumor sections (arrows) without blocking (A) and after (B) blocking of the α_v_ß_3_-integrin receptor using a specific antibody. Bottom row (C and D): the same tumor sections (arrows) scanned by optical fluorescence imaging after additional α_v_ß_3_-integrin-specific immunofluorescence stainings. C/A and D/B show identical tumor sections. SI = signal intensity, a.u. = arbitrary units. ^68^Ga-TRAP-(RGD)_3_ binding is significantly reduced after specific blocking of the α_v_ß_3_-integrin receptor (B vs. A). The immunofluorescence stainings confirmed the successful α_v_ß_3_-integrin receptor blocking in the autoradiography experiments (C and D). Note the complementary binding of the immunofluorescence staining and ^68^Ga-TRAP-(RGD)_3_ (C vs. A and D vs. B), indicating that binding of the primary α_v_ß_3_-integrin-specific antibody is effectively blocked by receptor-bound radiotracer.

CD31/α_v_β_3_-integrin fluorescent double stainings confirmed the predominantly endothelial α_v_β_3_-integrin expression in the investigated MDA-MB-231 human breast cancer xenografts (Fig 7). The combined autoradiography and immunofluorescence imaging experiments confirmed the target specificity of ^68^Ga-TRAP-(RGD)_3_ and demonstrated that binding of the primary anti-α_v_β_3_-integrin antibody is effectively blocked by receptor-bound radiotracer (Fig 8). Quantitative assessment in autoradiography revealed an intensity ratio of 2.71 ± 1.54 between unblocked and blocked tumor sections (p = 0.001). Detailed quantitative data for the autoradiography blocking experiments are provided in Table 3.

**Table 3:** Quantitative analysis of the autoradiography blocking experiments.

Tumor section no.	No blocking [intensity per area]	Blocked [intensity per area]	Ratio[unblocked/blocked]
1	17.24	10.57	1.63
2	15.32	11.94	1.28
3	33.51	6.48	5.17
4	20.78	8.09	2.57
5	14.85	11.51	1.29
6	41.75	25.95	1.61
7	41.19	20.22	2.04
8	17.64	3.46	5.10
9	15.32	8.84	1.73
10	20.61	10.29	2.00
11	65.41	16.37	4.00
12	16.55	11.39	1.45
13	98.26	17.08	5.75
14	16.06	7.29	2.20
15	28.20	14.01	2.01
16	35.94	19.55	1.84
17	12.04	5.77	2.01
18	15.71	3.10	5.07
**mean ± SD**^a^			2.71 ± 1.54^b^

^a^SD = standard deviation;

^b^p = 0.001 (unblocked vs. blocked)

## Discussion

In this experimental study, ^68^Ga-TRAP-(RGD)_3_-PET/CT allowed for the non-invasive monitoring of anti-angiogenic effects in a low α_v_ß_3_-integrin expression orthotopic human breast cancer model in mice, generating complementary and additional information to morphology-based tumor response assessments. ^68^Ga-TRAP-(RGD)_3_ binding was significantly reduced following VEGF inhibition, analogously to the significant suppression of α_v_ß_3_-integrin expression observed in the immunohistochemistry cohort. In untreated animals, ^68^Ga-TRAP-(RGD)_3_ uptake increased over the course of the experiment and α_v_ß_3_-integrin expression was found to be significantly higher.

Our observations are in accordance with a previous study investigating a ^68^Ga-labeled pegylated RGD dimer probe for monitoring the early anti-angiogenic effects of an endostatin therapy in heterotopic human lung cancer xenografts in mice [23]. Validated by ex vivo immunohistochemistry, the authors found the RGD radiotracer uptake to be significantly reduced following anti-angiogenic therapy, reflecting tumor response significantly earlier than ^18^F-FDG-PET. Contrary results were reported by Rylova et al. who studied ^68^Ga-NODAGA-RGD for the in vivo monitoring of a bevacizumab monotherapy in human squamous cell carcinoma xenografts in mice [12]. Despite a reduced α_v_ß_3_-integrin expression under VEGF inhibition, they observed an increased binding of ^68^Ga-NODAGA-RGD in the investigated A-431 xenografts. Rylova et al. therefore concluded that RGD radiotracer uptake might not necessarily reflect the changes in α_v_ß_3_-integrin expression on the molecular level. They hypothesized that bevacizumab may activate α_v_ß_3_-integrin, causing a higher affinity to ^68^Ga-NODAGA-RGD and consequently increased radiotracer uptake in vivo. Assuming a high-affinity state of α_v_ß_3_-integrin however, one would expect an increased binding of the primary anti-α_v_ß_3_-integrin antibody used in the immunohistochemical stainings and consequently false-high α_v_ß_3_-integrin levels. However, in line with our results, the authors found a reduced α_v_ß_3_-integrin expression in the therapy group. In addition, VEGF is known to indirectly activate α_v_ß_3_-integrin [9]. It therefore becomes unlikely that VEGF inhibition also causes α_v_ß_3_-integrin activation. The validation experiments in the present study revealed that binding of the primary anti-α_v_ß_3_-integrin antibody used in the immunohistochemical analysis is effectively blocked by receptor-bound radiotracer. Our results suggest that intraindividual immunohistochemical validation, i.e., α_v_ß_3_-integrin staining of tumors previously exposed to RGD radiotracers, may only be of limited value. We therefore conclude that the assessment of the α_v_ß_3_-integrin receptor status parallel to RGD PET imaging requires a dedicated immunohistochemistry animal cohort. Competitive blocking effects between receptor-bound radiotracer and the α_v_ß_3_-integrin-specific antibody may also be a possible explanation for the contrary observations reported by Rylova et al. [12].

Bevacizumab is the humanized form of mouse VEGF Mab A4.6.1 and therefore shows no cross-reactivity with circulating murine VEGF [24]. Nevertheless, ex vivo immunohistochemistry revealed significant anti-angiogenic effects of bevacizumab in the investigated MDA-MB-231 human breast cancer model in mice, as indicated by the suppression of endothelial marker CD31 and α_v_ß_3_-integrin expression under therapy. In line with these findings, significant anti-angiogenic effects of bevacizumab have also been observed in various other in vivo models of human cancer in murines [16, 25, 26]. Although not targeting murine VEGF, bevacizumab binds to tumor-derived human VEGF when applied in vivo [24]. As only human tumor cells and no stromal/endothelial cells were injected into the mammary fat pads of the nude mice in our study, the vasculature of the xenografts must be of murine origin [27]. However, as shown by Millauer et al. [28], the murine VEGF receptor 2 (= flk-1) shows a high affinity for human VEGF, resulting in upregulation of angiogenesis. Consequently, in vivo blocking of human VEGF using a human VEGF-specific monoclonal antibody (2C3) showed significant anti-angiogenic effects in MDA-MB-231 human breast cancer xenograft-bearing mice [29]. Therefore, bevacizumab exhibits significant anti-angiogenic effects in human tumor xenograft models in which angiogenesis is also driven by human VEGF [24]. Accordingly, Liang et al. found that the response of human cancer xenografts to bevacizumab is highly dependent on the grade of host stroma invasion and the presence of stroma-derived VEGF [30]. The authors demonstrated that human tumor xenografts with low murine stroma invasion show a better response to bevacizumab than those with high murine stroma invasion, as the angiogenic response predominantly relies on tumor-derived, human VEGF [30]. Bevacizumab thus blocked the tumor-derived but not the host-derived VEGF in the MDA-MB-231 human breast cancer xenografts investigated in the present study. This is in support of the defined study purpose, which was not to understand the host-specific VEGF response but to investigate a novel, α_v_ß_3_-integrin-targeted radiotracer for monitoring anti-angiogenic effects in a small animal model of human breast cancer. Our findings indicate that human, tumor-derived VEGF substantially triggers neovascularization in the investigated MDA-MB-231 human breast cancer model in mice and that a one-week bevacizumab monotherapy can be successfully applied to inhibit angiogenesis in this particular tumor model. However, it has to be acknowledged that residual, pro-angiogenic effects caused by uninhibited murine VEGF may still be present.

Our results confirm the applicability of ^68^Ga-TRAP-(RGD)_3_ in the investigated, low α_v_ß_3_-integrin expression tumor model. In a rodent model of myocardial infarction however, ^68^Ga-TRAP-(RGD)_3_ allowed for the assessment of α_v_ß_3_-integrin expression as imaging biomarker of myocardial repair, but, despite the higher target affinity in vitro [20], did not show a higher uptake than ^68^Ga-NODAGA-RGD or ^18^F-Galacto-RGD [31]. It can thus be postulated that the individual performance of different RGD radiotracers may vary depending on the investigated microstructures and tumor models. In the investigated MDA-MB-231 xenograft model, ^68^Ga-TRAP-(RGD)_3_ allowed for a robust and reproducible dual time-point in vivo imaging and generated moderate-to-good TBR.

α_v_ß_3_-integrin has recently gained attention as potential imaging target for the non-invasive in vivo characterization of the tumor microenvironment, and first clinical studies investigating radiolabeled RGD compounds in humans have been published [17, 32]. In particular, the in-human use of RGD-based radiotracers proved to be safe with tumor-specific uptake profiles [6, 33]. A recent clinical study even demonstrated the potential of combined functional parameters of ^68^Ga-NOTA-RGD- and ^18^F-FDG-PET/CT for the non-invasive molecular phenotyping of human breast cancer [34]. The combination of both radiotracers allowed for the distinction of breast cancer subtypes with regard to the receptor status (i.e., expression of the estrogen receptor, progesterone receptor, and Human Epidermal Growth Factor Receptor 2), although the underlying mechanisms still need to be identified. Therefore, the clinical translation and routine use of RGD-based hybrid imaging are likely to increase within the next years. The present study may provide a preclinical basis for future clinical studies investigating RGD-based hybrid imaging for monitoring therapy response to molecular cancer therapies. The expression of the α_v_ß_3_-integrin receptor and its distribution within the tumor (endothelial vs. cellular) should be assessed prior to clinical RGD-based hybrid imaging. This will help clinicians to interpret the imaging results as PET imaging is limited in its ability to distinguish between signal derived from tumor cells and signal derived from the tumor microenvironment. In the future, it will be of particular interest to investigate RGD PET-derived imaging biomarkers in correlation to clinical end points such as progression-free and overall patient survival. RGD-based hybrid imaging may allow for a non-invasive real-time molecular phenotyping of breast cancer under anti-angiogenic treatment, adding complementary molecular imaging biomarkers of therapy response to morphology-based and functional tumor response assessments. As part of multimodal imaging protocols, RGD-based hybrid imaging may help to identify patients who potentially benefit from targeted treatment regimens and therefore contribute to a tailored, personalized cancer therapy.

### Limitations

We acknowledge several limitations of the study. First, ^68^Ga-TRAP-(RGD)_3_ showed a renal clearance with impaired visualization of lesions adjacent to the urinary system, which is a known limitation of RGD radiotracers [6]. Accordingly, tumor regions with spillover from radiotracer in the urinary bladder were not included for the analysis. However, this is a common difficulty with many radiolabeled peptides, especially when labeled with short half-life radionuclides. Second, RGD radiotracers are small molecules with potential extravasation and may therefore also bind to α_v_ß_3_-integrin expressed by tumor cells. It cannot be quantified to what degree the RGD radiotracer is bound by the endothelium and to what degree the RGD radiotracer is bound by the tumor cells. However, we were able to show that α_v_ß_3_-integrin expression is predominantly reserved to the endothelium in the investigated tumor model and that the MDA-MB-231 tumor cells only exhibit marginal α_v_ß_3_-integrin expression [10]. Thus, our experiments confirmed that there is no relevant α_v_ß_3_-integrin expression in the extravascular compartment. Nevertheless, subtle enhanced permeability and retention-mediated effects as well as marginal radiotracer binding to the tumor cells cannot be completely excluded. However, these would be present to the same degree in both therapy and control group with no significant impact on whole-tumor TBR. Third, although overexpressed by angiogenic endothelium, α_v_ß_3_-integrins are expressed ubiquitously, e.g., in the gut and the liver, hence compromising the assessment of tumor TBR by elevated background signal and spillover [6]. Therefore, the respective tumor VOI needs to be selected accordingly. Fourth, the investigation of additional α_v_ß_3_-integrin-targeted radiotracers would allow for a side-by-side comparison of binding specificity and tumor signal in the examined MDA-MB-231 model of breast cancer.

## Conclusions

^68^Ga-TRAP-(RGD)_3_ proved to be applicable for the longitudinal in vivo monitoring of α_v_ß_3_-integrin expression as surrogate of neoangiogenesis in the investigated MDA-MB-231 xenografts in which α_v_ß_3_-integrin expression remains predominantly reserved to the endothelium. ^68^Ga-TRAP-(RGD)_3_ allowed for a robust and reproducible dual time-point in vivo imaging and, despite a low overall α_v_ß_3_-integrin expression, generated moderate-to-good TBR. It can be concluded that ^68^Ga-TRAP-(RGD)_3_ allows for the in vivo imaging of tumor models with low overall α_v_ß_3_-integrin expression.
